# Clinical and angiographic profile of left main coronary artery disease in patients with chronic coronary syndrome: a retrospective study

**DOI:** 10.1186/s43044-025-00615-5

**Published:** 2025-02-03

**Authors:** Abdulsalam Mahmoud Algamal, Mahmoud Abdelbadie Salem, Ahmed Ibrahim Bedier, Mohammed Salah A. Hussein, Mona Malek Abdelrahim, Shady Hussein Elhusseiny

**Affiliations:** Mansoura University, Al Mansurah, Egypt

**Keywords:** Coronary angiography, Coronary artery bypass grafting, Coronary artery disease, Coronary stenosis, Left main coronary artery disease, Percutaneous coronary intervention

## Abstract

**Background:**

Obstructive left main disease (LMD) is a challenging entity of coronary artery disease with variable patterns among different studies. We aimed to evaluate the prevalence, demographic, clinical, and angiographic profiles of LMD. We conducted a single-center retrospective study over a period of 10 years to screen all patients who underwent elective cardiac catheterization for chronic coronary syndrome. Of the 19,336 screened cases, 944 obstructive LMD patients were included as the patients' group. Age and sex-matched control groups included patients with normal coronary angiography and non-LMD.

**Results:**

Obstructive LMD had a prevalence of 4.9%, a mean age of around 60 years, and a male to female ratio of approximately 3:1. About 9.8% of LMD patients were < 50 years. Compared to males, females with LMD had significantly older age and increasing prevalence with age from 9.7% in patients < 50 years to 27.4% in patients > 70 years. LMD versus non-LMD patients had a significantly higher prevalence of diabetes mellitus, dyslipidemia, and number of stenotic coronary segments and arteries, and nonsignificant differences regarding smoking, hypertension, previous myocardial infarction, and ejection fraction. Ostial LMD had a prevalence of 2%, a mean age of around 58 years and 21% were females. In LMD patients, the most affected sites were the ostial/proximal left anterior descending artery and distal left main bifurcation. Bypass grafting surgery was the standard angiographic decision in LMD in 75.8% of cases, which was significantly higher than non-LMD. LMD patients revascularized surgically versus percutaneous treatment had significantly lower ejection fraction, significantly higher multivessel disease, and no significant differences regarding age, sex, hypertension, and diabetes mellitus.

**Conclusion:**

Obstructive LMD is a relatively common angiographic finding, with a higher prevalence among males around 60 years. In LMD, bypass grafting was the main revascularization strategy. We recommend integrating clinical characteristics, and noninvasive investigations as a predictive model of LMD.

## Background

Coronary artery disease (CAD) is a worldwide leading cause of morbidity and mortality with considerable socioeconomic load. Although cardiovascular mortality decreases in developed countries, developing countries still have a rising CAD burden [1]. Patients with chronic coronary syndrome (CCS) showed a wide spectrum of clinical scenarios including stress-induced angina, ischemia or angina with nonobstructive coronary arteries, non-acute patients after acute coronary syndromes, revascularization, ischemic or cardiometabolic heart failure, asymptomatic subjects diagnosed during coronary imaging for another reason [2]. The left main coronary artery (LMCA) supplies 75% to 100% of the left ventricular myocardium according to the coronary dominance [3]. Obstructive LMCA disease (LMD) is mainly atherosclerotic with variable clinical presentations ranging from asymptomatic to sudden death [4]. LMD is a high-risk and challenging entity of CAD with a highly variable prevalence among different studies ranging from 2.5% to 17.5% [5]. Invasive coronary angiography (CA) is still the diagnostic gold standard of CAD, however, some clinical, electrocardiographic (ECG), and noninvasive testing may differentiate LMD from other CAD subsets [6]. Revascularization of LMD patients should be decided by a multidisciplinary heart team taking into consideration multiple cardiac and non-cardiac comorbidities [7]. LMD patients are heterogeneous regarding complexity, coexisting coronary lesions, gender differences, and age distribution [8]. Most studies in Egypt and the Middle East/North Africa region investigated LMD, while assessing other CAD presentations or angiographic patterns. Other studies focused on LMD revascularization strategies, special clinical situations, and prognostic outcomes. El-Moselhy et al. [9] assessed CAD risk factors among elderly Egyptian patients and estimated LMD prevalence. Aldosari et al. [10] investigated CAD patterns among Saudi patients and evaluated the LMD prevalence and its associations with age. Leon et al. [11] assessed percutaneous coronary interventions (PCI) of LMD as one of the complex interventions and its impact on outcomes. Ayman et al. [12] investigated PCI outcomes of unprotected LMD among Egyptian patients. Daoulah et al. [13] compared the outcomes of PCI versus coronary artery bypass grafting (CABG) in patients with and without emergent LMD. Daoulah et al. [14] studied the impact of preoperative intra-aortic balloon pump on PCI outcomes among LMD patients. Few local studies screened for the prevalence, risk profile, and angiographic patterns of LMD patients. This retrospective study aimed to evaluate LMD patients regarding prevalence, risk profile, and angiographic characteristics and their age and gender differences.

## Methods

This is a single-center retrospective study screening all patients who underwent first-time elective CA for CCS evaluation in cath. Lab. in Mansoura Medical Specialized Hospital over a period of 10 years from January 2012 to December 2021. To overcome the interobserver variability, angiographic films were analyzed by 2 authors in addition to the written angiographic report, and a final agreement on the severity of coronary stenosis was reached. All patients with Obstructive LMD were selected as the patients` group. Two age and sex-matched control groups were recruited; the first group included 500 patients with normal CA and the second group included 500 patients with non-left main CAD (non-LMD) defined as obstructive stenosis in one or more epicardial coronary arteries not involving the LMCA. Obstructive CAD was defined as > 50% stenosis in the LMCA and/or > 70% stenosis in the epicardial coronary arteries, and normal CA was defined as a smooth outline and normal flow in the epicardial vessels [6]. We excluded patients with nonsignificant LMCA stenosis, presentation with acute coronary syndromes, and previous coronary revascularization. We reported clinical data, cardiovascular risk factors, angina class, ECG findings, ejection fraction (EF), and echocardiographic wall motion abnormalities (WMA). Identifying a patient as having single, double, or multivessel disease was based on the presence of obstructive CAD in one or more of the 3 coronary arteries and their branches. Ostial isolated LMD was defined as > 50% stenosis of the proximal LMCA not extending > 3 mm from the coronary origin [15]. We used the 16-segment model of the coronary arterial tree as proposed by the American Heart Association in 1975, edited by the Arterial Revascularization Therapies Study I and II, and adopted in the Synergy between Percutaneous Coronary Interventions with TAXUS and Cardiac Surgery (SYNTAX) score [16]. Calculation of the SYNTAX score was done online at http://www.syntaxscore.com. The angiographic decision of revascularization by PCI or CABG was made by the operator of the case.

## Statistical analysis

Data collection, tabulation, and analysis were performed using the Statistical Package of Social Science (SPSS, IBM, Inc, Chicago; USA) version 26 for Windows. The normality of quantitative data was tested using the Kolmogrov-Smirnov test and expressed as mean ± SD. Categorical data were expressed as percentages and frequency. Independent sample T and Mann–Whitney tests were used for intergroup comparison of parametric and nonparametric continuous data respectively. The Chi-square test or Fisher’s exact test was used for comparing two categorical data. Probability (P < 0.05) was statistically significant.

## Results

**Fig. 1:**
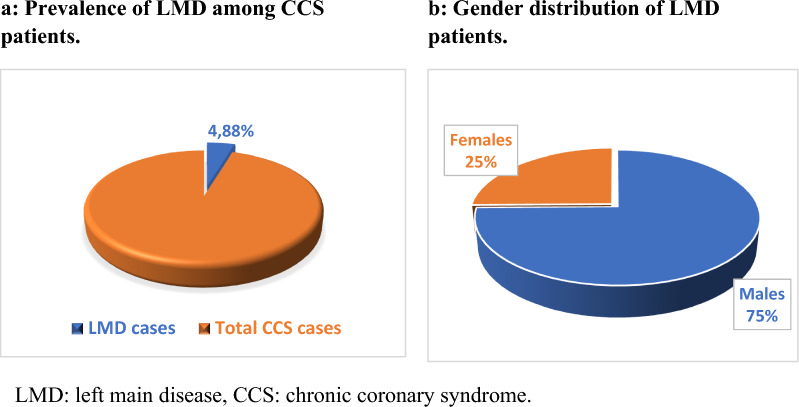
**a** Prevalence of LMD among CCS patients. **b**: Gender distribution of LMD patients. LMD: left main disease, CCS: chronic coronary syndrome

Of the 19,336 screened CCS cases, we found 944 obstructive LMD patients with a prevalence of 4.9% (Fig. 1a). The mean age of LMD patients was 60.37 ± 7.617 years with males representing 74.8% of cases (Fig. 1b). 9.8% of LMD patients were < 50 years. The percentage of female LMD patients increased with age from 9.7% in patients < 50 years to 27.4% in patients > 70 years. Smoking, diabetes mellitus (DM), hypertension, and dyslipidemia affected 59.4%, 42.6%, 47.7%, and 47.6% of LMD cases respectively, indicating the high prevalence of classical cardiovascular risk factors. Compared to patients with normal CA, LMD patients had significantly lower EF and significantly higher prevalence of smoking, DM, hypertension, dyslipidemia, angina class, ECG abnormalities, and WMA. LMD versus non-LMD patients had a significantly higher prevalence of DM, dyslipidemia, angina class, and left bundle branch block, significantly less normal ECG, and no significant difference regarding smoking, hypertension, ST-T changes, Q waves, EF, and WMA. There was no significant difference among all groups regarding hepatitis C seropositivity, family history of CAD, and atrial fibrillation (Table 1).

**Table 1:** Characteristics of all patients

	LMD group (no = 944)	Non-LMD group (no = 500)	Normal CA group (no = 500)	P1 value	P2 Value
Age (years)	60.37 ± 7.617	60.18 ± 7.848	61.14 ± 7.485	0.532	0.202
Sex					
Male	706(74.8%)	379(75.8%)	381(76.2%)	0.672	0.554
Female	238(25.2%)	121(24.2%)	119(23.8%)	Female	
Hepatitis C seropositivity	256(27.1%)	137(27.4%)	142(28.4%)	0.909	0.604
Smoking	561(59.4%)	301(60.2%)	126(25.2%)	0.776	< 0.001*
Diabetes mellitus	402(42.6%)	184(36.8%)	113(22.6%)	0.033*	< 0.001*
Hypertension	450(47.7%)	240(48.0%)	205(41.0%)	0.905	0.015*
Dyslipidemia	449(47.6%)	206(41.2%)	210(42.0%)	0.021*	0.043*
Family history of CAD	186(19.7%)	103(20.6%)	101(20.2%)	0.685	0.822
Angina class >2	573(60.7%	273(54.6%	96(19.2%	0.025*	< 0.001*
Atrial fibrillation	15(1.6%)	7(1.4%)	10(2.0%)	0.780	0.569
ECG					
Normal	46(4.9%)	68(13.6%)	201(40.2%)	< 0.001*	< 0.001*
ST-T changes	631(66.8%)	318(63.6%)	241(48.2%)	0.217	
Left BBB	149(15.8%)	49(9.8%)	58(11.6%)	0.002*	0.031*
Q wave	118(12.5%)	65(13.0%)	0(0.0%)	0.786	< 0.001*
Ejection fraction	55.48 ± 8.378	55.77 ± 8.481	58.35 ± 9.055	0.532	< 0.001*
Wall motion abnormalities	398(42.2%)	185(37.0%)	80(16.0%)	0.057	< 0.001*

LMD = left main disease, CA = coronary angiography, CAD = coronary artery disease, ECG = electrocardiogram, BBB = bundle branch block, P1 = LMD versus non-LMD groups, P2 = LMD versus normal CA groups, * = significant

LMD versus non-LMD patients showed a significantly higher number of stenotic coronary segments and vessels, SYNTAX score, left anterior descending artery (LAD) affection and angiographic decision of CABG, significantly less single vessel disease and right coronary artery (RCA) affection and no significant difference regarding left circumflex artery (LCX) affection and previous ST-segment elevation myocardial infarction (STEMI) (Table 2).

**Table 2:** Characteristics of LMD versus non-LMD patients

	LMD group (no = 944)	Non-LMD group (no = 500)	*P* value
Old myocardial infarction			
Anterior	93(9.9%)	51(10.2%)	0.903
Inferior	25(2.6%)	15(3.0%)	
LAD disease	851(90.1%)	379(75.8%)	< 0.001*
LCX disease	538(57.0%)	285(57.0%)	0.998
RCA disease	291(30.8%)	181(36.2%)	0.038*
number of stenotic segments	3.23 ± 1.083	2.48 ± 0.850	< 0.001*
Number of stenotic vessels			
Single	295 (31.25%)	211 (42.2%)	< 0.001*
Two	481 (50.95%)	232 (46.4%)	
Three	168 (17.8%)	57 (11.4%)	
SYNTAX score	22.08 ± 5.388	11.64 ± 7.446	< 0.001*
Angiographic decision			
PCI	228(24.2%)	445(89.0%)	< 0.001*
CABG	716(75.8%)	55(11.0%)	

LMD = left main disease, LAD = left anterior descending coronary artery, LCX = left circumflex coronary artery, RCA = right coronary artery, SYNTAX = Synergy between PCI with TAXUS and Cardiac Surgery, PCI = percutaneous coronary intervention, CABG = coronary artery bypass grafting, * = significant

**Fig. 2:**
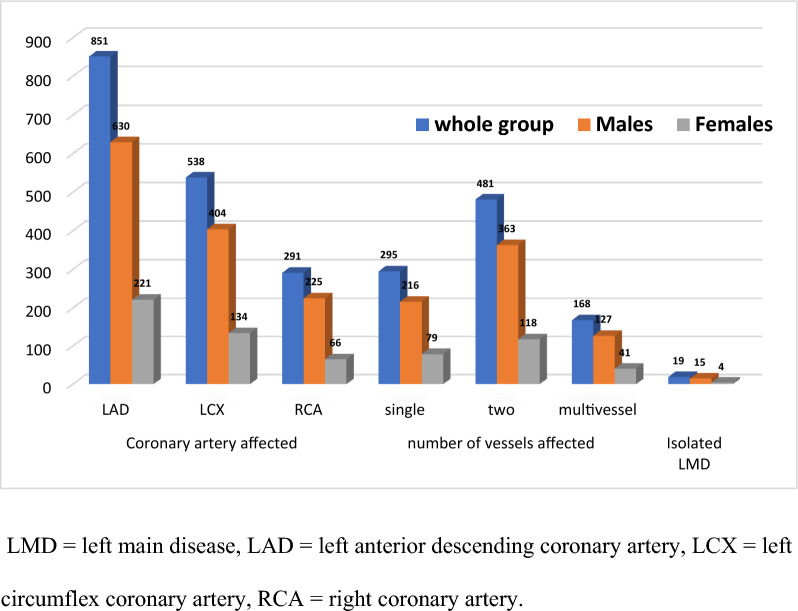
Angiographic patterns of LMD patients: LMD = left main disease, LAD = left anterior descending coronary artery, LCX = left circumflex coronary artery, RCA = right coronary artery

Diffuse LMD affected 3.4% of cases and the distal left main bifurcation in 91%, whereas ostial LMD affected 2% of cases. In patients with distal LMD, Medina classifications (1:1:1), (1:1:0), (1:0:1), and (1:0:0) affected 33.1%, 40.1%, 23.2%, and 3.6% cases respectively. Among LMD patients, LAD was the most affected vessel in 90.1% of cases, while LCX and RCA were affected in 57% and 30.8% of cases respectively. Single, double, and multivessel disease affected 31.3%, 51%, and 17.7% of LMD patients respectively (Fig. 2).

In LMD patients, male versus female patients showed a significantly higher prevalence of smoking and inferior myocardial infarction (MI), significantly lower age, dyslipidemia, and anterior MI, and no significant difference regarding DM, hypertension, angina class, EF, WMA, number of stenotic coronary segments and vessels, angiographic patterns, SYNTAX score, and decision of revascularization (Fig. 2 and Table 3). LMD patients revascularized by CABG versus PCI had significantly lower EF, significantly higher prevalence of WMA, MI, LAD, and LCX disease, multivessel disease, and SYNTAX score, and no significant difference regarding age, sex, DM, hypertension, angina class, number of stenotic coronary segments, and RCA disease (Table 4).

**Table 3:** Characteristics of male versus female patients with left main disease:

	Males (no = 706)	Females (no = 238)	*P* value
Age (years)	59.90 ± 7.915	61.76 ± 6.474	< 0.001*
Smoking	522 (73.9%)	39 (16.4%)	< 0.001*
Diabetes mellitus	293 (41.5%)	109 (45.8%)	0.246
Hypertension	343 (48.6%)	107 (45.0%)	0.333
Dyslipidemia	322 (45.6%)	127 (53.4%)	0.038*
Angina class >2	418 (59.2%)	155 (65.1%)	0.106
Ejection fraction	55.34 ± 8.225	55.89 ± 8.822	0.381
Wall motion abnormalities	302 (42.8%)	96 (40.3%)	0.510
Old myocardial infarction			
Anterior	63 (8.9%)	30 (12.6%)	0.041*
Inferior	23 (3.3%)	2 (0.8%)	
LAD disease	630 (89.2%)	221 (92.9%)	0.105
LCX disease	404 (57.2%)	134 (56.3%)	0.804
RCA disease	225 (31.9%)	66 (27.7%)	0.232
number of stenotic segments	3.23 ± 1.080	3.21 ± 1.095	0.852
Number of stenotic vessels			
Single	216 (30.6%)	79 (33.2%)	0.756
Two	363 (51.4%)	118 (49.6%)	
Three	127 (18.0%)	41 (17.2%)	
SYNTAX score	22.17 ± 5.508	21.82 ± 5.018	0.375
Angiographic decision			
PCI	166 (23.5%)	62 (26.1%)	0.429
CABG	540 (76.5%)	176 (73.9%)	

LAD = left anterior descending coronary artery, LCX = left circumflex coronary artery, RCA = right coronary artery, SYNTAX = Synergy between PCI with TAXUS and Cardiac Surgery, PCI = percutaneous coronary intervention, CABG = coronary artery bypass grafting, * = significant

**Table 4:** Characteristics of patients with left main disease according to the angiographic decision:

	PCI (no = 228)	CABG (no = 716)	*P* value
Age (years)	60.70 ± 7.298	60.27 ± 7.718	0.458
Sex			
Male	166 (72.8%)	540 (75.4%)	0.429
Female	62 (27.2%)	176 (24.6%)	
Smoking	133 (58.3%)	428 (59.8%)	0.699
Diabetes mellitus	98 (43.0%)	304 (42.5%)	0.889
Hypertension	98 (43.0%)	352 (49.2%)	0.104
Dyslipidemia	104 (45.6%)	345 (48.2%)	0.499
Angina class ˃2	130 (57.0%)	443 (61.9%)	0.191
Ejection fraction	57.91 ± 7.506	54.70 ± 8.497	< 0.001*
Wall motion abnormalities	45 (19.7%)	353 (49.3%)	< 0.001*
Old myocardial infarction			
Anterior	12 (5.3%)	81 (11.3%)	0.008*
Inferior	3 (1.3%)	22 (3.1%)	
LAD disease	165 (72.4%)	686 (95.8%)	< 0.001*
LCX disease	107 (46.9%)	431 (60.2%)	< 0.001*
RCA disease	66 (28.9%)	225 (31.4%)	0.481
number of stenotic segments	3.18 ± 1.082	3.24 ± 1.084	0.464
Number of stenotic vessels			
Single	86 (37.7%)	209 (29.2%)	0.007*
Two	115 (50.4%)	366 (51.1%)	
Three	27 (11.8%)	141 (19.7%)	
SYNTAX score	20.96 ± 4.067	22.44 ± 5.701	< 0.001*

PCI = percutaneous coronary intervention, CABG = coronary artery bypass grafting, LAD = left anterior descending coronary artery, LCX = left circumflex coronary artery, RCA = right coronary artery, SYNTAX = Synergy between PCI with TAXUS and Cardiac Surgery, * = significant

## Discussion

Given the large area of jeopardized myocardium, obstructive LMD was associated with important therapeutic and prognostic implications and represented an independent predictor of heightened morbidity and mortality among CAD patients [4]. The heart team approach for LMD management was emphasized by current guidelines considering the patients' preferences, anatomic complexity, and surgical risk [7]. We aimed to investigate clinical and angiographic LMD characteristics over a decade among Egyptian patients. Providing clinical background and angiographic analysis of LMD would help in the prediction, prognostication, and management of LMD.

## Prevalence of LMD

We showed that LMD prevalence was 4.9% among CCS patients. Previous studies of LMD prevalence showed high variability. Lower prevalence was seen in some studies as 2.2% [5], 2.4% [17], 3% [18], and 3.4% [19]. Comparable prevalence was shown by other studies as 4.8% [20]. Higher prevalence was shown by other studies as 8.7% [21], 10.3% [10] and 10.5% [22]. LMD prevalence was higher among high-risk patients with acute coronary syndromes [23]. The variability of LMD prevalence among different studies may be explained by variable risk profiles and clinical presentation of patients.

## Age and gender distribution of LMD

In our study, the mean age of presentation of LMD was around 60 years, which was higher than some studies as 52 years [21] and 55 years [8]. Comparable age around 60 years was described in previous studies [24–26]. A higher age of presentation was seen in some studies as around 65 years [27–29] and 72 years [30]. High-risk patients presented with STEMI, non-STEMI (NSTEMI), and cardiac arrest showed higher age of presentation around 67.5 years [31]. Some studies showed no significant association of LMD with age [10].

In our study, 74.8% of LMD patients were males. Concordant results were shown by previous studies as 70% [32], 75% [26, 33], around 78% [29, 34], around 84% [25, 31], and 90.5% [21]. This may be partly explained by much thinner LMCA in females even with similar body surface area [35].

## Clinical characteristics of LMD versus non-LMD patients

Our study showed that traditional cardiovascular risk factors were highly prevalent among LMD patients with smoking, hypertension, DM, and dyslipidemia affecting 59.4%, 47.7%, 42.6%, and 47.6% respectively. Different studies showed variable risk profiles among LMD patients. Smoking prevalence was 36.9% [19], 55.1% [24] and 71% [36], hypertension incidence was 50% [22] and around 62% [17], DM prevalence was 29% [26], 33% [28], 42% [25], and 57.8% [36], whereas the prevalence of dyslipidemia was shown to be 30.8% [21], 46.9% [25], around 71% [17]. In high-risk patients presented with STEMI, non-STEMI, and cardiac arrest who underwent emergency PCI of culprit LMCA lesions, Bajaj et al. [31] showed that smoking, DM, hypertension, and dyslipidemia affected 53%, 24%, 59%, and 53% of patients respectively.

In our study, LMD versus non-LMD patients showed a significantly higher prevalence of DM, dyslipidemia, and angina class and nonsignificant differences regarding smoking, and hypertension. Virani et al. [37] indicated similar risk profiles of LMD and non-LMD patients including DM, smoking, hypertension, hypercholesterolemia, and obesity. Female gender and older age were associated with a higher LMD probability after post hoc analysis of the Initial Invasive or Conservative Strategy for Stable Coronary Disease (ISCHEMIA) trial [38]. Kostkiewicz et al. [39] showed that LMD compared to non-LMD patients had a significantly higher prevalence of male gender, DM, hypertension, and typical angina, significantly lower family history of CAD, and no significant difference regarding the age of presentation, dyslipidemia, and smoking. Biswas et al. [8] showed that LMD versus non-LMD patients had a statistically significant higher prevalence of male gender and DM, a statistically significant lower family history of CAD, and no significant difference regarding the age of presentation, hypertension, dyslipidemia, and smoking. Askari et al. [25] showed that LMD compared to non-LMD patients had a statistically significant higher prevalence of male gender, dyslipidemia, and family history of CAD, and no statistically significant difference regarding the age of presentation, hypertension, and DM. Gehani et al. [18] showed that LMD compared to non-LMD patients had a statistically significant lower prevalence of hypertension, dyslipidemia, and smoking, and no significant difference regarding sex, DM, and family history of CAD. In high-risk NSTEMI patients, Claver et al. [23] showed that LMD compared to non-LMD patients had statistically significant higher age, DM, and heart failure and no significant difference regarding gender distribution, and angina class. Similarly, in NSTEMI patients, Kosuge et al. [40] showed that LMD compared to non-LMD patients had statistically significant higher age, smoking, and DM, whereas there was no significant difference regarding sex, dyslipidemia, hypertension, and family history of CAD.

In our study, previous MI affected 12.5% of LMD patients (9.9% anterior and 2.6% inferior MI) with no significant difference between LMD and non-LMD patients. History of MI was associated with a higher probability of LMD using post hoc analysis of the ISCHEMIA trial [38]. Previous studies showed that among LMD patients, previous MI was identified in around 8.5% [27], 14% [24], 18% [41] and 34.7% [30]. Compared to non-LMD, some previous studies showed significantly higher previous MI in LMD patients [40], whereas other studies showed nonsignificant differences [18, 39].

## ECG and echocardiographic characteristics of LMD versus non-LMD patients

In our study, LMD versus non-LMD patients showed significantly less normal ECG, significantly higher left bundle branch block, and no significant difference regarding ST-T changes, EF, and WMA. Khawaja et al. [4] showed that ECG changes in LMD patients presented with CCS may be subtle as non-specific ST-T changes and the diagnosis may be further complicated by atypical symptoms. In high-risk NSTEMI patients, Claver et al. [23] showed that LMD compared to non-LMD patients showed no significant difference regarding ECG changes.

In our study, LMD patients had a mean EF of around 55% with no significant difference versus non-LMD patients regarding EF and WMA. Previous studies showed that the mean EF in LMD patients was 48% [19], 55% [42], 57.4% [41], and 60% [27]. Some previous studies showed significantly lower EF in LMD than non-LMD patients [23], whereas other studies showed a nonsignificant difference [8, 18]. Concordant to our results, Biswas et al. [8] showed that LMD versus non-LMD patients had no significant difference regarding WMA.

## Cardiovascular risk factors in male versus female patients with LMD

Our study showed that among LMD patients, male versus female patients showed significantly higher smoking and inferior MI, significantly lower age, dyslipidemia, and anterior MI, and no significant difference regarding DM, hypertension, angina class, EF, WMA, number of stenotic coronary segments and vessels, angiographic patterns, SYNTAX score, and decision of revascularization. In our study, 9.8% of LMD patients were < 50 years. The percentage of female patients increased with age from 9.7% in patients < 50 years to 27.4% in patients > 70 years. Comparing male to female patients with LMD showed contradictory results in previous studies. Sheiban et al. [32] showed that female patients were significantly older than male patients and had significantly higher DM, hypertension, and clinical presentation with acute coronary syndrome. Wang et al. [35] showed that males had significantly higher smoking compared to females with no significant differences regarding age, hypertension, DM, dyslipidemia, family history of CAD, MI, EF, number of stenotic coronary arteries, and the pattern of involvement of the LMCA. McEntegart et al. [34] showed that females had significantly higher DM, hypertension, family history of CAD, EF, significantly less distal LMD and SYNTAX score, and no significant difference regarding age, smoking, clinical presentation, and angiographic decision to treat by PCI or CABG.

## Angiographic patterns of LMD patients

We showed that among LMD patients, ostial LMD affected 2% of cases with a mean age of 58 years and 21% were females. Srinivas et al. [5] showed that the incidence of ostial LMD varied between 0.1% and 2.7% of LMD cases with coexisting disease in other coronary arteries in most cases. Previous studies showed a variable prevalence of ostial LMD as 3.3% [24], 4.7% [20], and 4 to 6% [3]. Contrary to our study, most previous studies showed a higher prevalence of ostial LMD in female patients [5, 18], with lower mean age of presentation and LMCA ostium as the main site [5]. Persson et al. [43] showed that PCI was more common in ostial LMD patients.

In our study, LAD was the most affected vessel (90.1%) among LMD patients with single, double, and multivessel disease affecting 31.3%, 51%, and 17.7% of cases respectively. Teniente-Valente et al. [17] showed significant stenosis of the LAD, LCX, and RCA in 80.9%, 71.4%, and 66.6% of LMD cases respectively. Biswas et al. [8] showed that LAD was the most affected artery in LMD patients, whereas Giannoglou et al. [20] showed that RCA significant stenosis was more common in LMD patients.

In our study, the distal bifurcation was the most affected site in 91% of LMD patients. Shabeer et al. [21] showed that LMD affects the distal end with proximal LAD and LCX involvement in most cases and is usually associated with multivessel disease. Previous studies showed the involvement of distal bifurcation in 67.4% [19], 70% [28], and 80.5% [29]. The higher affection of distal bifurcation of LMCA may be due to local hemodynamic forces and endothelial shear stress which had a determining role in the location of atherosclerosis [4].

We showed that among patients with distal LMD, Medina classifications (1:1:1), (1:1:0), (1:0:1), and (1:0:0) affected 33.1%, 40%, 23.2%, and 3.7% cases respectively. Rao [24] showed that the patterns of Medina classification of distal LMD were (1:1:1), (1:1:0), (1:0:1), and (1:0:0) in 49.8%, 25.1%, 14.3%, and 10.8% respectively. In high-risk patients undergoing emergency PCI for culprit lesions in the LMCA, Bajaj et al. [31] showed that distal bifurcation lesion was the most prevalent anatomy and Medina classification (1:1:1), (1:1:0), (1:0:1), and (1:0:0) affected 51%, 25%, 8%, and 16% respectively. We showed that the mean SYNTAX score among LMD patients was 22.08 ± 5.38 which was significantly higher than non-LMD patients. In previous studies, the mean SYNTAX score in LMD patients was around 26.5 ± 9.3 [28]. Gao et al. [41] showed that the mean SYNTAX score in LMD patients who underwent PCI guided by intravascular ultrasound versus angiography was 28.4 ± 13.8 and 34.0 ± 15.9 respectively.

## Angiographic decisions of LMD patients

In our study, CABG was the standard method of revascularization in LMD patients, and the decision was made in 75.8% of cases, which was significantly higher than in non-LMD patients. The heart team should tailor the revascularization strategy considering clinical and angiographic factors with increasing use of PCI depending on the advances in coronary imaging, physiological assessment, and coronary devices [44]. Persson et al. [43] showed that the proportion of LMD patients treated with PCI increased from 7% in 2005 to 34% in 2015. Studies comparing the percutaneous versus surgical interventions showed mixed results [44]. Shah et al. [45] showed that studies comparing the superiority of CABG versus PCI was especially marked in non-LMD, but not in LMD patients. In the elderly, Dąbrowski et al. [44] showed similar effects of PCI and CABG concerning mortality and quality of life suggesting that PCI is an important alternative to CABG. Persson et al. [43] showed that CABG was the revascularization method in 84% of LMD patients and was associated with lower mortality and major adverse cardiovascular and cerebrovascular events.

Our study showed that LMD patients revascularized by CABG versus PCI had significantly lower EF and significantly higher WMA, MI, LAD, LCX disease, multivessel disease, and SYNTAX score. There were no significant differences regarding age, sex, DM, hypertension, angina class, number of stenotic coronary segments, and RCA disease. Although DM was higher in our patients treated by CABG versus PCI, the difference was statistically insignificant. This finding was discordant to most previous studies. Dąbrowski et al. [44] concluded that CABG remains the treatment of choice for diabetic patients with LMD. Similarly, the 2024 ESC management guidelines for CCS showed that in diabetic patients with multivessel disease, CABG was recommended over PCI to improve symptoms and outcomes [2]. However, other studies showed similar outcomes of PCI versus CABG in diabetic patients with LMD [46].

Steigen et al. [33] showed no significant difference between LMD patients treated by PCI or CABG regarding age, sex, DM, hypertension, smoking, SYNTAX score, EF, clinical presentation, and distal LMD. Yu et al. [26] concluded that LMD patients treated by CABG had significantly higher previous MI and multivessel disease, significantly lower dyslipidemia, EF, ostial LMD, and single vessel disease, while there was no significant difference regarding age, sex, smoking, hypertension, and DM. Persson et al. [43] showed that LMD patients treated by CABG had significantly higher smoking, DM, and significantly lower age, hyperlipidemia, previous MI, and ostial LMD.

## Conclusion

Obstructive LMD is a relatively common angiographic finding with a prevalence of 4.9% in patients who underwent elective CA for CCS evaluation. LMD patients had a mean age of around 60 years and male to female ratio of approximately 3 to 1. Compared to males, females with LMD had significantly higher age and their prevalence increased with age. LAD and distal left main bifurcation were the most affected sites in LMD patients, whereas ostial LMD affected 2% of cases. CABG was the standard method of revascularization in LMD patients. We recommend prospective large-scale studies and registries integrating clinical, noninvasive investigations, coronary angiographic parameters, especially intracoronary imaging, and pressure assessment to develop a predictive model of obstructive LMD and to assess outcomes and morbidity of LMD patients.

## Limitations

The study's main limitations included the single-center retrospective design, the visual angiographic assessment, and the selection bias of control groups.

## Data Availability

No datasets were generated or analyzed during the current study.

## List of abbreviations

CA: Coronary angiography
CABG: Coronary artery bypass grafting
CAD: Coronary artery disease
CCS: Chronic coronary syndrome
DM: Diabetes mellitus
ECG: Electrocardiographic
EF: Ejection fraction
LAD: Left anterior descending artery
LCX: Left circumflex artery
LMCA: Left main coronary artery
LMD: Left main coronary artery disease
MI: Myocardial infarction
PCI: Percutaneous coronary interventions
RCA: Right coronary artery
STEMI: ST-segment elevation myocardial infarction
SYNTAX: Synergy between Percutaneous Coronary Interventions with TAXUS and Cardiac Surgery
WMA: Wall motion abnormalities
